# Nutrient contents predict the bamboo‐leaf‐based diet of Assamese macaques living in limestone forests of southwest Guangxi, China

**DOI:** 10.1002/ece3.6297

**Published:** 2020-04-28

**Authors:** Yuhui Li, Guangzhi Ma, Qihai Zhou, Youbang Li, Zhonghao Huang

**Affiliations:** ^1^ Key Laboratory of Ecology of Rare and Endangered Species and Environmental Protection (Guangxi Normal University) Ministry of Education Guilin China; ^2^ Guangxi Key Laboratory of Rare and Endangered Animal Ecology Guangxi Normal University Guilin China; ^3^ School of Life Sciences South China Normal University Guangzhou China

**Keywords:** Assamese macaques, bamboo‐leaf‐based diet, limestone forests, *Macaca assamensis*, Nutrient contents

## Abstract

Determining the nutrient factors influencing food choice provides important insight into the feeding strategy of animals, which is crucial for understanding their behavioral response to environmental changes. A bamboo‐leaf‐based diet is rare among mammals. Animals’ food choice and nutritional goals have been explained by several frameworks; however, the influence of nutrients on food choice in bamboo‐leaf‐based macaques is not yet available. Assamese macaques (*Macaca assamensis*) inhabiting limestone forests are characterized by such a bamboo‐leaf‐based diet, predominantly consuming young leaves of *Bonia saxatilis*, a shrubby, karst‐endemic bamboo. We studied the feeding behavior of one group of Assamese macaques using instantaneous scan sampling in limestone forests of the Guangxi Nonggang National Nature Reserve in southwest Guangxi, China. We compared the nutrient content of staple food and nonfood items and examine the role of key nutrients in the food selection of macaques. Our results showed that young leaves of bamboo *B. saxatilis* contained more water, crude protein, phosphorus, and less tannin than nonfood items. Furthermore, staple foods contained a higher content of water and less content of calcium than nonfood items. More specifically, quantities of water, crude protein, calcium, and phosphorus in food items were critical factors affecting feeding time on a specific plant item. Our results suggest that young bamboo leaves could meet macaques’ required protein and water intake, while enabling them to maintain their mineral balance, consequently facilitating to maintain the primates’ bamboo‐leaf‐diet in the limestone forest. Our findings confirm the effects of nutrient contents in food choice of Assamese macaques, highlighting the importance of the nutrient contents in maintaining their bamboo‐based diet and the need to increase the knowledge on their nutritional strategy adapted to the bamboo‐dominated diet inhabiting the unique limestone habitat.

## Graphical abstract

Using behavior and nutriology methods, we found that young bamboo leaves provided resident macaques required protein, water, and mineral intake, likely enabling animals to maintain bamboo‐leaf‐based diet with combining behavioral adjustment. Our results provide an important insight into the nutrition strategy of primates in responses to limestone forest, consequently further deepening our understanding in why animals maintain a bamboo‐leaf‐based diet from the nutrient dimension.

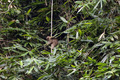

## 1 INTRODUCTION

Determining the nutrients influencing food choice can provide an important insight into the feeding strategy of primates, which is crucial to understand their behavioral responses to environmental changes (Altmann, 1998; Felton, Felton, Lindenmayer, & Foley, 2009). Different food species and plant parts vary significantly in nutritional content, and the nutrients provided by a single food type are often suboptimal, forcing primates to develop generalist feeding strategies to meet their nutritional goals (Altmann, 2009; Donati et al., 2017; Eppley et al., 2017; Felton, Felton, Lindenmayer, et al., 2009; Ganzhorn et al., 2017; Rothman, Raubenheimer, & Chapman, 2011). In general, nutritional goals are explained by following five major models (Felton, Felton, Lindenmayer, et al., 2009; Zhao et al., 2013): (1) the energy maximization model states that primates are committed to maximizing their energy (Hixon, 1982; Schoener, 1971); and (2) the nitrogen (protein) maximization model states that primates prefer foods rich in protein (Eppley et al., 2017; Ganzhorn et al., 2017; Miton, 1979); both models are supported by observational evidence that consumption of a specific food item is positively correlated with its energy and protein content. Moreover, the limitations of the dietary fiber model (3) (Milton, 1979) and the avoidance of plant secondary metabolites (PSMs) model (4) (Freeland & Janzen, 1974) both state that primates avoid foods with a high fiber and/or PSMs content, as shown by the negative correlation between feeding intensity and the food item's fiber and PSMs content. In addition, the nutrient balancing model (5) states that primates try to achieve nutrient balance, rather than focusing on maximizing specific nutrient intake (Raubenheimer & Simpson, 2004). However, recent studies have illustrated those models mentioned above do not apply uniformly for animals (DeGabriel et al., 2014; Donati et al., 2017; Ganzhorn et al., 2017; Wallis et al., 2012), implying complexity and site‐specific dependence of food choice in primates (Ganzhorn, 1992; Ganzhorn et al., 2017).

Among the nutritional factors influencing food choice, protein and fiber have been considered vital determinants (Amato & Garber, 2014; DeGabriel et al., 2014; Dröscher, Rothman, Ganzhorn, & Kappeler, 2016; Ganzhorn et al., 2017; Ma, Liao, & Fan, 2017; Zhao et al., 2013). Protein concentration varies in different plant species and plant parts (Richard, 1985). In general, younger leaves have higher protein content than other plant parts such as fruits or mature leaves (Amato & Garber, 2014; Nie et al., 2019; Richard, 1985). Many primates tend to predominantly select protein‐rich foods (Chapman & Chapman, 2002; Ganzhorn et al., 2017), as shown in *Colobus guereza* (Fashing, Dierenfeld, & Mowry, 2007), *Colobus polykomos* (Dasilva, 1994), *Ateles chamek* (Felton, Felton, Wood, et al., 2009) and *Lepilemur leucopus* (Dröscher et al., 2016). As young leaves mature, the cell wall thickens and protein content decreases, resulting in an increase of cellulose, hemicellulose, and lignin (collectively called “fiber”) (Amato & Garber, 2014; Nie et al., 2019; Yeager, Silver, & Dierenfeld, 1997). Due to a lack of specific appropriate enzymes in primates to hydrolyze fiber, consuming large amount of high‐fiber foods increases their digestion time and consequently decreases feeding benefit (Barboza et al., 2010; Barboza, Parker, & Hume, 2009). Therefore, primates tend to prefer high‐protein foods over high‐fiber food items (Ganzhorn et al., 2017; Nakagawa, 2003; Wallis et al., 2012).

Water is an indispensable medium for thermoregulation, as evaporation requests a large amount of water (Houser, Crocker, & Costa, 2005). For example, in baboons (*Papio hamadryas ursinus*), water replenishment decreases body temperature by nearly 3°C within one hour (Brain & Mitchell, 1999). Thus, water is considered determinant in driving food choice, particularly for primates living in arid habitats where free standing water sources are rare (Scott, 2005; Wu, Huang, Yuan, Deng, & Zhou, 2011). In these arid environments, primates normally obtain water from their diet, showing a preference for water‐rich items (Scott, 2005). For example, in limestone forests where almost no surface water is available, white‐headed langurs (*Trachypithecus leucocephalus*) (Huang, 2002; Huang, Wei, Li, Li, & Sun, 2003) and rhesus macaques (*Macaca mulatta*) (Tang et al., 2016) obtain most of their water intake from their diet, an adaptation which arose to cope with water deficiency in the surrounding environment (Huang, 2002; Scott, 2005). A similar strategy has been found in hybrid marmosets (*Callithrix spp.*) who prefer to consume exudates from *Anadenanthera peregrina* containing high water content (Francisco et al., 2017) and Peruvian spider monkeys (*Ateles chamek*) who primarily consume *Ficus boliviana* to obtain the water they need (Felton, Felton, Wood, et al., 2009).

Minerals such as calcium (Ca), magnesium (Mg), and phosphorus (P) are essential for mammals, and mineral deficiency would result in abnormal growth (Irwin, Raharison, Chapman, Junge, & Rothman, 2017; Reynolds, Pascual‐Garrido, Lloyd, Lyons, & Hobaiter, 2019; Robbins, 1983; Zhang & Watanabe, 2010; Zhao et al., 2013). Particularly, Ca deficiency can cause osteoporosis (Agata et al., 2013) and muscle spasms (Szent‐Györgyi, 1975), as well as impact neuronal transmission (Simons, 1988), hormone secretion (Draznin, 1988), and acid‐base balance (Semel et al., 2019). Thus, primates consciously replenish their minerals by consuming high‐Ca food items, such as mature leaves and soil. For instance, black howler monkeys (*Alouatta pigra*) feed on a large quantity of mature leaves to offset the absence of figs (Ca‐rich) in their diet (Behie & Pavelka, 2012). Although minerals are essential nutrients, in excess and/or imbalance, they can be linked to adverse effects (Nie et al., 2015; Robbins, 1983; Stephen, 1991). In general, a Ca:P intake of 1:1–2:1 is appropriate for mammals (Nie et al., 2015). An excess of certain minerals could interfere with the absorption of the other minerals (Robbins, 1983), even leading to disease (Khan et al., 2016; Uribarri & Calvo, 2013).

Bamboo‐leaf‐based diet is rare among mammals (Mekonnen et al., 2018), with only six primate species/populations known to heavily depend on bamboo leaves: the golden bamboo lemur (*Hapalemur aureus*) (78% of bamboo in diet), the gray bamboo lemur (*H. griseus*) (72% of bamboo in diet), and the greater bamboo lemur (*H. simus*) (95% of bamboo in diet) in Madagascar (Overdorff, Strai, & Telo, 1997; Tan, 1999); the Bale monkeys (*Chlorocebus djamdjamensis*) (76.7% of bamboo in diet) in Ethiopia (Mekonnen, Bekele, Fashing, Hemson, & Atickem, 2010); the golden monkeys (*Cercopithecus mitis kandti*) in Uganda (52.4% of bamboo in diet) (Twinomugisha, Chapman, Lawes, Worman, & Danish, 2006) and the Assamese macaques (*M. assamensis*) (71.2% of bamboo in diet) in southwest China (Huang et al., 2015). Those bamboo‐based primates depend heavily on bamboo likely for the purpose of seeking for high protein concentrations in the food, despite leading to high plant secondary metabolites intakes, as shown in *Hapalemur* spp. (Eppley et al., 2017). Similarly, giant pandas (*Ailuropoda melanoleuca*), who depend almost exclusively on bamboo items (Wei, Fan, & Hu, 2020; Wei et al., 2015), seasonally migrate in accordance with phenologies of their dietary bamboo species, and their migrations are associated with diet switching sequences, being characterized by maximizing the proportion of protein in macronutrient intakes and by decreasing fiber consumptions (Nie et al., 2019). Moreover, pandas maintain an exceptionally low daily energy expenditure as behavioral response to their bamboo‐based diet (Nie et al., 2015).

Assamese macaques (*M. assamensis*) are partly distributed in limestone forests of southwest Guangxi, China (Zhang, 1997). This area is covered by limestone and other soluble carbonate rocks with a thin layer of Ca‐rich soil (Tu & Yang, 1995; Yuan, 1994). Special carbonate substrates characterize the many distinctive features of limestone forests. Limestone hills typically have lower plant biomass but higher vegetation diversity than the nonkarst regions, with a large number of endemic calciphilous plant species (Ji, Li, & Deng, 2009; Li, He, et al., 2019; Tu & Yang, 1995). Moreover, although rainfall is abundant during the rainy season, rainwater flows away directly along fractures and caverns, resulting in the absence of surface water (Cao, Yuan, & Pan, 2003; Yuan, 1994). Previous studies have shown that Assamese macaques living in limestone forests predominantly consume young leaves of *Bonia saxatilis*, a karst‐endemic bamboo (71.2% of annual record with a monthly range from 48.8% to 94.1%) (Huang et al., 2015). Detailed data on the nutritional strategy of this bamboo‐leaf‐based diet are not yet available. In this study, we present the first available data on the nutritional strategy of karst‐dwelling Assamese macaques and compare the nutritional contents between food and nonfood items in their diet. We aim to determine the nutritional factors driving their bamboo‐leaf‐based diet by testing following predictions:

1. The nitrogen (protein) maximization model hypothesizes that primates prefer foods high in protein (Mattson, 1980). Assamese macaques consume young bamboo leaves throughout the year (Huang et al., 2015), and young bamboo leaves are richer in protein and less in fiber than other plant parts (Nie et al., 2019; Richard, 1985). Therefore, we expected that this food item should have higher protein content than nonfood parts, as the protein maximization model predicted.
2. Limestone forests are characterized by a lack of surface water (Cao et al., 2003; Yuan, 1994), resulting in primates meeting their hydration requirements principally through their diet (Huang, 2002; Huang et al., 2003; Tang et al., 2016). A previous study also found this group consumed more young leaves in the afternoon, probably to replenish water (Li, Huang, Zhou, Ma, & Huang, 2019). Therefore, we expected that the water content in Assamese macaques’ foods should be higher than that of nonfood items.
3. Limestone forests are characterized by high Ca, with a large number of calciphilous plants (Ford & Williams, 2007; Ji et al., 2009; Li, He, et al., 2019; Tu & Yang, 1995). Although Ca is important, excess and/or imbalance consumption blocks the absorption of other minerals and may also cause diseases (Khan et al., 2016; Robbins, 1983). Therefore, we expected that the Ca content in food items would be lower than that in nonfood items.

## 2 METHODS

### 2.1 Study site and animals

This work was conducted at the limestone seasonal forest in the Guangxi Nonggang National Nature Reserve (22° 29′ 25″ N, 106° 53′ 33″ E), southwest Guangxi, China. The altitudinal range of the site varies between 400 and 600 m above sea level (Guangxi Forestry Department, 1993). The top ten dominant plant families are Euphorbiaceae, Moraceae, Ebenaceae, Tiliaceae, Poaceae, Meliaceae, Sterculiaceae, Verbenaceae, Rubiaceae, and Sapindaceae, respectively (Huang et al., 2015).

Data on climate, including minimum and maximum temperature and rainfall, were recorded during the study period using a hyetometer and a thermometer. The total rainfall was 1,055 mm, and the mean monthly minimum and maximum temperatures were 14.9°C and 26.5°C, respectively. More detailed climatic information is provided in an earlier study conducted on the same site (Huang et al., 2015).

One group of Assamese macaque was studied, including 14 individuals (2 adult males, 6 adult females, 6 juveniles) at the starting of the study. By the end of the study, the group had increased to 16 members, after the birth of 2 infants.

### 2.2 Behavioral data and samples collection

Behavioral data were collected using an instantaneous scan sampling method (Altmann, 1974), employing 5‐min scans followed by 10‐min intervals, between September 2012 and August 2013 after a 3‐month habituation period from June to August 2012. When the individuals manually or orally manipulated a food part, we recorded them as feeding. Meanwhile, the plant species and items eaten by each observed individual were recorded. Feeding behavior and the food species and parts during the scanning 10‐min intervals were also recorded via ad libitum sampling (Altmann, 1974). The data from ad libitum sampling were used to compose a food species list, but were not used for analysis of dietary composition. A detailed procedure of the behavioral data collection carried out in this study is described in Huang et al. (2015) and Li, Huang, et al. (2019).

Plant samples were collected from May to August 2014, based on the data of dietary composition and vegetation investigation on this group, which used nine plants as staple foods (Huang et al., 2015). We collected 111 samples of 37 items (3 samples per item) from 25 plant species, including 7 staple foods species and 18 species of the top 20 most abundant plants species (Table 1). We collected most of food samples from the plant individuals consumed by the sampled macaques. However, a few samples were collected from other plants located in near feeding plants, due to inaccessibility or unavailability of the original plant. We collected young bamboo leaves in the same feeding patch where macaques spent their feeding time. For the nonfood species, we randomly selected trees for sampling. In addition, we collected the mature bamboo leaves from those dropped by macaques in their feeding sites. For each sample, we collected >250 g of plant items and sealed them airtight bags labeled with the species name, part of the plant, collection date, and location. We admit that there could be limitation caused by collecting plant samples discarding the temporal variations in the nutrient content; however, this study undoubtedly provides a general pattern of nutritional strategy of these limestone‐dwelling Assamese macaques.

**Table 1:** Food and nonfood samples collected for nutritional analysis

Species	Plant parts*, **	Food/Nonfood**, **
*Bonia saxatilis*	YL	Y
	ML	*N*
*Indosasa angustata*	YL	Y
*Trachelospermum brevistylum*	ML	Y
*Tetrastigma planicaule*	FR	Y
*Caryota ochlandra*	FR	Y
*Streblus indicus*	YL, FR	Y
*Spondia lakonensis*	FR	Y
*Diospyros chunii*	YL, ML, FR	*N*
*Diospyros siderophylla*	YL, ML	*N*
*Ficus microcarpa*	YL, ML	*N*
*Garcinia paucinervis*	YL, ML	*N*
*Streblus indicus*	ML	*N*
*Vitex kwangsiensis*	YL, ML	*N*
*Cephalomappa sinensis*	YL, ML	*N*
*Ficus hispida*	YL, ML	*N*
*Deutzianthus tonkinensis*	YL, ML, FR	*N*
*Clausena lansium*	ML	*N*
*Helicia formosana*	ML	*N*
*Boniodendron minius*	ML	*N*
*Walsura robusta*	ML	*N*
*Garuga pinnata*	ML	*N*
*Excentrodendron tonkinense*	ML	*N*
*Lysidice rhodostegia*	ML	*N*
*Garuga forrestii*	ML	*N*
*Indosasa angustata*	ML	*N*

* YL, young leaves; ML, mature leaves; FR, fruits.

** Y, species/items used for food by macaques; N, species/items not used for food by macaques.

### 2.3 Nutrient content analysis experiment

We measured the water content of each sample using the weight method (Standardization Administration of the People's Republic of China, 2011a): All samples were measured for fresh weight (W_t_) and then were dried to a constant weight (W_c_) at 65°C to determine their water content, expressed as a percentage of water weight using following formula: $Water\left(\%\right)=\frac{W_{t}-W_{c}}{W_{t}}\times 100\%$ (Standardization Administration of the People's Republic of China, 2011a). After calculating water content, we ground all samples into a fine powder and sifted them through a 40 Mesh sieve before storing them in sealable plastic bags until further analysis. The analysis of the remaining nutrients was based on the dry matter of the sample. We measured absolute dry matter by drying samples at 103°C ± 2°C (Standardization Administration of the People's Republic of China, 2013). We expressed the dry matter contents as the percentages of the dry sample weights in the weight before drying. Crude protein was determined using the Kjeldahl method (Standardization Administration of the People's Republic of China, 2011b); crude fiber was measured using the acid‐base digestion method (Standardization Administration of the People's Republic of China, 2003a); crude fat was weighted using the Soxhlet extraction method (Standardization Administration of the People's Republic of China, 2003b); crude ash was examined using the dry ash method (Chinese Academy of Forestry, 1999a); and tannin was measured using spectrophotometry (Standardization Administration of the People's Republic of China, 2008a). The contents of crude protein, crude fiber, crude fat, crude ash, and tannin were presented as percentages of specific nutrient content weights in dry matter. In addition, mineral contents (Ca, Mg, and P) were determined using spectrophotometry and presented as microgram per gram of dry matter (μg/g) (Chinese Academy of Forestry, 1999b; Standardization Administration of the People's Republic of China, 2008b; 2011b). All samples were tested twice to ensure accuracy (samples were re‐measured when the replicated results varied by >5%) at the Key Laboratory of Ecology of Rare and Endangered Species and Environmental Protection (Guangxi Normal University), Ministry of Education, China.

### 2.4 Data analysis

Following Huang et al. (2015), we determined feeding time per various food items using following method. First, we divided the number of sampled macaques eating specific items within each scan by the total amount of members noted in that scan and then divided it by the percentage of macaques feeding to calculate the proportion of feeding time on specific items in the given scan. Then, we calculated hourly feeding time on specific items, using the average of values for all scans within an hour. To correct for bias potentially resulted from uneven scan records throughout the day, we averaged hourly data to express daily feeding time on specific food items. We then averaged daily data within each month to determine monthly feeding time on specific food items. We expressed monthly feeding time devoted to specific items as the average proportion across the relevant months. Finally, we determined annual feeding time on specific plant species by averaging all monthly averages.

To improve linearity, numeric variables such as mineral contents (Ca, Mg, and P) were log_10_(X)‐transformed (Li et al., 2015; Xu et al., 2017), whereas the variables expressed in percentages including water, dry matter, crude protein, crude fiber, crude fat, crude ash, and tannin contents were logit(X)‐transformed (Warton & Hui, 2011). The feeding time were logit(X + 0.00001)‐transformed, because the raw data of the feeding time for those nonfood species were zero. All variables were tested using one‐sample Kolmogorov–Smirnov to examine the normality of the data before proceeding to further statistical analysis. The results suggested that all data did not deviate from normality. Following Huang et al. (2017), we constructed generalized linear mixed models (GLMMs) to examine the differences in nutritional content between food and nonfood items. We set nutritional contents as response variables, plant types (food or nonfood) as fixed factor, and plant parts as random factor in the model. Then, we performed ANOVA when comparing the models with and without fixed factors to determine the effects of fixed factors on each dependent variable. Plant type (fixed factor) was regarded as a factor that significantly impacted the goodness of fit of the model when the probability value (*p*‐value) was <.05, suggesting that response variable existed markedly difference between food and nonfood species.

Following Li et al. (2015) and Xu et al. (2017), we constructed GLMMs to investigate the effects of nutritional content on feeding time per individual plant species. We set the feeding time on plant species as response variable, the nutritional contents as fixed factors, and the plant parts as random factors in the model. In total, we obtained 2^10^–1 = 1,023 models from all possible combinations of the 10 fixed factors, and conducted multimodel inference and model averaging based on information theory (Akaike's information criterion corrected for small sample sizes, AICc). In the multimodel inference, models were ranked based on AICc values and the model with the lowest AICc value was considered the highest ranked model. We selected the most highly supported models where the difference of AICc values between each model and the highest ranked model were ≤2 (ΔAIC ≤2) and calculated Akaike weights (W*_i_*) of each most highly supported model. In model averaging, each predictor variable was evaluated for its relative importance (W*_ip_*) by summing the W*_i_* values of all the models including specific variable to determine the impact of predictor variables on the response variable (Burnham & Anderson, 2002). In addition, the model‐averaged regression coefficients (*β*) and the 95% confidence intervals of *β* (95% CI) of each predictor were also calculated. When *β* = 0, we considered that the matching variable should be excluded from that model (Burnham & Anderson, 2002). The GLMMs were constructed using the *lmer* function of the *lme4* package (Bates, Mächler, Bolker, & Walker, 2015); the model averaging was performed using *dredge* and *model.avg* function of the *MuMIn* package (Bartoń, 2019). All analyses were conducted using R v3.6.1 (R Development Core Team, 2019). All tests were two‐tailed, with significance levels of 0.05.

## 3 RESULTS

### 3.1 Nutrient characteristics of *Bonia saxatilis*

Young leaves of *B. saxatilis* contained 89.3 ± 3.6% water, 90.4 ± 0.1% dry matter, 32.1 ± 0.2% crude protein, 12.5 ± 0.1% crude fiber, 5.1 ± 0.1% crude fat, 10.9 ± 0.0% crude ash, and < 0.1 ± 0.0% tannin. Moreover, Ca in *B. saxatilis* was 775.8 ± 11.6 μg/g, with 292.1 ± 4.1 μg/g Mg, and 5,907.6 ± 22.2 μg/g P. Young leaves of *B. saxatilis* significantly differed from nonfood species in certain nutrients, being embodied in higher content of water (χ^2^ = 12.624, *df* = 1, *p* < .001), crude protein (χ^2^ = 12.412, *df* = 1, *p* < .001), P (χ^2^ = 5.010, *df* = 1, *p* = .025), and lower tannin (χ^2^ = 9.966, *df* = 1, *p* = .002) (Tables 2 and 3).

**Table 2:** Comparisons of nutrient contents for the bamboo leaves and nonfood items

Response variable	Explanatory variable	Estimated values	Standard error	t
Water	Intercept	0.723	0.210	3.445
	Food species	1.182	0.307	3.844
Dry matter	Intercept	2.665	0.042	63.690
	Food species	−0.428	0.233	−1.835
Crude protein	Intercept	−2.055	0.159	−12.960
	Food species	1.103	0.292	3.785
Crude fiber	Intercept	−1.584	0.289	−5.471
	Food species	0.115	0.497	0.231
Crude fat	Intercept	−3.678	0.164	−22.493
	Food species	0.858	0.503	1.705
Crude ash	Intercept	−2.652	0.181	−14.688
	Food species	0.623	0.395	1.557
Tannin	Intercept	−3.985	0.327	−12.181
	Food species	−6.040	1.822	−3.316
Ca	Intercept	2.993	0.085	35.151
	Food species	0.004	0.155	0.029
Mg	Intercept	2.493	0.046	54.020
	Food species	0.004	0.091	0.040
P	Intercept	3.241	0.104	31.170
	Food species	0.399	0.178	2.245

**Table 3:** Variations in nutrient contents of the bamboo young leaves and nonfood items (Mean ± *SD*)

Nutrient contents	*Bonia saxatilis*	Nonfood species			χ^2^ (*df* = 1)	*p*
		Young leaves	Mature leaves	Fruits		
Water (%)	89.3 ± 3.6	71.9 ± 4.9	58.4 ± 7.5	71.7 ± 1.0	12.624	<.001*, **
Dry matter (%)	90.4 ± 0.1	93.2 ± 1.3	93.5 ± 1.3	93.2 ± 0.1	3.406	.065
Crude protein (%)	32.1 ± 0.2	14.3 ± 4.3	11.9 ± 2.5	8.0 ± 0.3	12.412	<.001*, **
Crude fiber (%)	12.5 ± 0.1	12.0 ± 6.4	20.6 ± 6.8	24.8 ± 6.7	0.031	.861
Crude fat (%)	5.1 ± 0.1	2.4 ± 1.4	3.4 ± 2.0	2.1 ± 1.2	2.374	.123
Crude ash (%)	10.9 ± 0.0	6.9 ± 4.3	8.8 ± 2.3	4.7 ± 1.1	2.274	.132
Tannin (%)	< 0.1 ± 0.0	5.6 ± 6.2	4.4 ± 4.3	1.9 ± 2.0	9.966	.002*, **
Ca(μg/g)	775.8 ± 11.6	837.7 ± 409.4	1,368.4 ± 244.0	911.7 ± 70.5	< 0.001	.990
Mg(μg/g)	292.1 ± 4.1	297.2 ± 81.0	373.7 ± 38.4	271.4 ± 88.9	< 0.001	.997
P(μg/g)	5,907.6 ± 22.2	2,620.9 ± 1,118.4	1,269.2 ± 507.8	1945.2 ± 48.9	5.010	.025*

* *p* < .05.

** *p* < .01.

### 3.2 Differences in nutrient content between food and nonfood items

Compared with nonfood items, food plant species had a higher content of water (χ^2^ = 10.700, *df* = 1, *p* = .001), but a lower content of Ca (χ^2^ = 4.816, *df* = 1, *p* = .028). However, the contents of dry matter (χ^2^ = 0.425, *df* = 1, *p* = .514), crude protein (χ^2^ = 2.185, *df* = 1, *p* = .139), crude fiber (χ^2^ = 0.301, *df* = 1, *p* = .583), crude fat (χ^2^ = 2.630, *df* = 1, *p* = .105), crude ash (χ^2^ = 0.630, *df* = 1, *p* = .428), tannin (χ^2^ = 3.551, *df* = 1, *p* = .060), Mg (χ^2^ = 1.695, *df* = 1, *p* = .193), and P (χ^2^ = 0.260, *df* = 1, *p* = .610) did not differ between food and nonfood items (Tables 4 and 5).

**Table 4:** Differences in nutrient contents of food and nonfood species, with the young bamboo leaves being included in analyses

Response variable	Explanatory variable	Estimated values	Standard error	t
Water	Intercept	0.661	0.240	2.760
	Food species	0.639	0.186	3.430
Dry matter	Intercept	2.655	0.055	48.397
	Food species	−0.053	0.110	−0.487
Crude protein	Intercept	−2.183	0.337	−6.480
	Food species	0.285	0.176	1.620
Crude fiber	Intercept	−1.584	0.266	−5.953
	Food species	−0.144	0.236	−0.610
Crude fat	Intercept	−3.705	0.181	−20.498
	Food species	0.526	0.254	2.073
Crude ash	Intercept	−2.716	0.236	−11.496
	Food species	0.210	0.198	1.061
Tannin	Intercept	−3.985	0.370	−10.774
	Food species	−1.597	0.850	−1.878
Ca	Intercept	3.031	0.138	21.983
	Food species	−0.319	0.142	−2.253
Mg	Intercept	2.498	0.045	55.060
	Food species	−0.057	0.047	−1.216
P	Intercept	3.222	0.121	26.695
	Food species	0.046	0.092	0.497

**Table 5:** Nutrient contents of predominated food and nonfood items (Mean ± *SD*), with the young bamboo leaves being included in analyses

Nutrient contents	Young leaves		Mature leaves		Fruits		χ^2^ (*df* = 1)	*p*
	Main food	Nonfood	Main food	Nonfood	Main food	Nonfood		
Water (%)	91.0 ± 2.5	71.9 ± 4.9	60.2 ± 7.9	58.4 ± 1.7	73.4 ± 9.6	71.7 ± 1.0	10.700	.001**, **
Dry matter (%)	91.6 ± 1.8	93.2 ± 1.3	96.5 ± 0.1	93.5 ± 1.3	92.4 ± 1.4	93.2 ± 0.1	0.425	.514
Crude protein (%)	29.2 ± 4.2	14.3 ± 4.3	13.2 ± 0.5	11.9 ± 2.5	6.5 ± 3.1	8.0 ± 0.3	2.185	.139
Crude fiber (%)	11.1 ± 2.0	12.0 ± 6.4	14.9 ± 1.7	20.6 ± 6.8	20.7 ± 11.4	24.8 ± 6.7	0.301	.583
Crude fat (%)	4.3 ± 1.1	2.4 ± 1.4	6.4 ± 0.2	3.4 ± 2.0	4.3 ± 4.0	2.1 ± 1.2	2.630	.105
Crude ash (%)	11.7 ± 1.1	6.9 ± 4.3	8.0 ± 0.3	8.8 ± 2.3	5.1 ± 2.9	4.7 ± 1.1	0.630	.428
Tannin (%)	< 0.1 ± 0.0	5.6 ± 6.2	7.4 ± 1.0	4.4 ± 4.3	1.7 ± 1.3	1.9 ± 2.0	3.551	.060
Ca (μg/g)	395.2 ± 538.3	837.7 ± 409.4	1,489.5 ± 15.1	1,368.4 ± 244.0	942.9 ± 206.1	911.7 ± 70.5	4.816	.028*
Mg (μg/g)	218.9 ± 103.6	297.2 ± 81.0	398.2 ± 2.2	373.7 ± 38.4	279.6 ± 69.6	271.4 ± 88.9	1.695	.193
P (μg/g)	5,413.6 ± 698.6	2,620.9 ± 1,118.4	725.0 ± 85.3	1,269.2 ± 1507.8	1536.3 ± 684.5	1945.2 ± 48.8	0.260	.610

* *p* < .05.

** *p* < .01.

### 3.3 Effects of nutrient contents on feeding time

The five most highly supported models (ΔAIC ≤2) contained nine variables: Ca, Mg, P, crude protein, water, dry matter, crude fat, crude ash, and crude fiber. Five variables were shared among all highly supported models, including Ca, Mg, P, crude protein, and water. However, the value of the Akaike weight (W*_i_*) of models exhibited high model selection uncertainties (W*_i_* = 0.14–0.32, Table 6).

**Table 6:** The top five linear regressions models (lm) (ΔAIC ≤2) investigating the effects of nutrient content on the feeding effort of Assamese macaques

Variable	Model 1	Model 2	Model 3	Model 4	Model 5
Ca	●	●	●	●	●
Mg	●	●	●	●	●
P	●	●	●	●	●
Crude protein	●	●	●	●	●
Water	●	●	●	●	●
Dry matter		●			
Crude fat			●		
Crude ash				●	
Crude fiber					●
AICc	145.00	145.49	146.48	146.59	146.62
ΔAIC	0.00	0.50	1.49	1.60	1.62
W*_i_*	0.32	0.25	0.15	0.14	0.14

Abbreviations: ●, variable included in the model; AICc, Akaike's information criterion corrected for small sample sizes; W*_i_* (Akaike weights), the probability that a model is best given the particular set of models considered; ΔAIC, difference between specific model and the most high‐ranked one.

The model averaging showed that water, crude protein, Ca, and P were significant factors affecting feeding time in Assamese macaques (W*_ip_* = 0.96–1.00, Table 7). Model‐averaged 95% confidence intervals of parameter estimates (*β*) of these variables also excluded zero. The factors mentioned above were also included into the five most highly supported models (ΔAIC ≤ 2; Table 6). Feeding time increased (*β* > 0) with the water, crude protein, and Ca, but decreased (*β* < 0) with P. Although Mg had a relatively important influence on feeding time (W*_ip_* = 0.99), their model‐averaged 95% confidence interval of the regression coefficient (*β*) contained zero. Other variables including dry matter, crude fiber, crude fat, crude ash, and tannin showed relatively less importance (W*_ip_* = 0.05–0.39), with 95% confidence intervals of the regression coefficient (*β*) overlapped zero.

**Table 7:** Summary of model averaging based on lm models (1,023 models) using nutrient factors to explain the feeding time on specific food of Assamese macaques

Variable	*β*	SE	z	p	95% CI		W*_ip_*
					Min	Max	
**Water**	**4.775**	**1.218**	**3.784**	**<.001**	**2.302**	**7.248**	**1.00**
Dry matter	0.931	1.430	0.625	.532	−1.987	3.849	0.39
**Crude protein**	**3.517**	**1.182**	**2.861**	**.004**	**1.108**	**5.926**	**0.96**
Crude fiber	−0.591	0.613	0.925	.355	−1.842	0.661	0.27
Crude fat	0.621	0.534	1.115	.265	−0.471	1.713	0.24
Crude ash	0.245	1.110	0.212	.832	−2.018	2.508	0.26
Tannin	−0.119	0.225	0.511	.610	−0.574	0.337	0.05
**Ca**	**4.729**	**2.023**	**2.256**	**.024**	**0.621**	**8.837**	**0.99**
Mg	−10.745	5.987	1.737	.082	−22.868	1.377	0.99
**P**	**−8.385**	**2.843**	**2.847**	**.004**	**−14.158**	**−2.611**	**1.00**

Note

Model‐averaged 95% confidence intervals excluded zero listed in bold.

Abbreviations: 95% CI, the 95% confidence intervals for *β*; W*_ip_*, relative variable importance; *β*, model‐averaged regression coefficients.

## 4 DISCUSSION

Our results showed that the young bamboo leaves had higher protein content than nonfood parts; moreover, crude protein was a significant predictor included in the five most highly supported models, with high relative importance (W*_ip_* = 0.96) and showed a positive correlation with feeding time (*β* > 0), supporting our first prediction. Such preferences have also been observed in other primates, such as *Lepilemur leucopus* (Dröscher et al., 2016),  *Colobus guereza* (Fashing et al., 2007), *Ateles chamek* (Felton, Felton, Wood, et al., 2009), and *C. polykomos* (Dasilva, 1994). This tendency reflects the fact that primates normally have a protein threshold intake which they need to exceed to remain healthy (approximately 1 g/kg of body weight per day); below this threshold, individuals are vulnerable to negative nitrogen balance, which can eventually result in death (Richard, 1985). Numerous primates have a preference for protein‐rich foods, favoring protein content in food selection (Chapman & Chapman, 2002; Eppley et al., 2017; Ganzhorn et al., 2017). For example, the eastern black‐and‐white colobus (*C. guereza*) choose specific leaves where protein content is ≥14% of dry matter (Fashing et al., 2007). Although we have no data on the specific protein threshold of our group, the protein content of all young leaves they used (29.2%) was higher than that of many other folivores primates, such as *Presbytis senex* (11%), *Semnopithecus entellus* (10%–16%) (Hladik, 1977), and *T. leucocephalus* (16%–20%) (Tang, 2004), indicating the Assamese macaques’ need for protein to be even higher. Such high protein content may be due to their bamboo‐leaf‐based diet. As mentioned previously, young leaves of karst‐endemic bamboo *B. saxatilis* accounts for 71.2% of the dietary composition of Assamese macaques in limestone forests (Huang et al., 2015). Compared with mature leaves or other plant parts, young leaves are the major source of protein (Amato & Garber, 2014; Ganzhorn et al., 2017; Richard, 1985). Furthermore, our results also show that the protein content in *B. saxatilis* reaches up to 32.1% of dry matter, not only higher than in nonfood items (14.3%) but also exceeding the average for all the young leaves they eat (29.2%), partly explaining the higher protein level found in their diet. On the other hand, fiber is avoided by many primates (Chapman & Chapman, 2002; Fashing et al., 2007; Nakagawa, 2003; Yeager et al., 1997). Surprisingly, our study found that fiber content was excluded from all highly supported models, without difference between food and nonfood items, suggesting that fiber content had no significant effect on the food choice of Assamese macaques. In other words, they did not consciously avoid high‐fiber foods, contrary to most other primates. Indeed, they may not need to deliberately avoid fiber, as young leaves (the staple food item) contain less fiber than mature leaves and fruits (Table 3). Therefore, our results suggest that Assamese macaques aim to maximize their protein intake, regardless of the fiber content on food choice as their staple foods provide abundant young leaves which are low in fiber.

The water content in food items was higher than those in nonfood items, with the highest relative importance (1.00) when modeling the role of nutrients in food choice, supporting our second prediction. Because water acts as a thermal buffer in the evaporation process, it is essential in the processes of thermoregulation (Houser et al., 2005). Primates selected succulent foods to meet their water requirement (*Callithrix spp.* (Francisco et al., 2017) and *Ateles chamek* (Felton, Felton, Wood, et al., 2009)). It is also known that mountain gorillas (*Gorilla beringei*) drink water from streams or extend their lower lip during rainfall to meet their water requirements (Rothman, Dierenfeld, Hintz, & Pell, 2008). In this study, the Assamese macaques’ main food items had a higher water content, which could be explained by the absence of surface water in limestone forests. The soil layer in the karst area is thinner, weakening the water retention capacity of the soil (Cao et al., 2003). In addition, many underground karst‐caves in limestone forests allow the rainwater to directly flow away along the fractures and cavern, resulting in a severe shortage of free surface water in these regions (Tu & Yang, 1995; Yuan, 1994). Thus, Assamese macaques seem to only use succulent foods to meet their daily water requirements. The young bamboo leaves had higher content of water than the nonfood items (Table 3), as revealed by the difference between main food items and nonfood parts (Table 5). Indeed, we never observed any individual drinking free water throughout the duration of this study. Moreover, our results showed that young leaves eaten contained more water than mature leaves and fruits used by the macaques. In our previous study of this group, we found that they consumed more young leaves during afternoon, possibly to rapidly meet their water need after the midday heat (Li, Huang, et al., 2019). Similarly, other sympatric primates also prefer to select succulent plants to cope with the lack of water in limestone forests (Huang, 2002; Huang et al., 2003; Tang et al., 2016). Therefore, choosing foods with a higher water content may be an important way for the Assamese macaques to maintain thermoregulatory balance, consequently contributing to their adaptation to the unusual limestone habitats.

Calcium was included in the five most highly supported models and was less abundant in food items than in nonfood items, which supports our third prediction. In accordance with other studies, our results found that Ca was a significant predictor of food choice (Behie & Pavelka, 2012; Francisco et al., 2017). Many primates favor Ca‐rich foods, such as the black howler monkey (*A. Pigra*) who feed on more mature leaves (Behie & Pavelka, 2012), and marmosets (*Callithrix spp.*) who predominantly feed on the gum of *Anadenanthera peregrina* (Francisco et al., 2017). Assamese macaques, however, prefer to feed on relatively low‐Ca foods in limestone forests, which could be related to the high Ca content in plant species found in this habitat. It is generally acknowledged that both soil and plants contain a higher Ca concentration in limestone forests than in nonkarst regions (Cao et al., 2003; Ford & Williams, 2007; Ji et al., 2009; Li, He, et al., 2019; Tu & Yang, 1995; Yuan, 1994). Living in such a Ca‐rich environment, Assamese macaques tend to choose staple foods characterized by a low Ca content, likely to minimize Ca intake. However, Assamese macaques actively replenished their P content: Although there was no significant difference in P between staple foods and nonfood items, P in young leaves of *B. saxatilis* was significantly higher than that of nonfood species, which could be explained by their mineral balance strategy. Previous study demonstrated that the Ca:P ratio was crucial for bone mass density and strength of mammals, with a recommended range from 1:1 to 2:1 (Calvo & Tucker, 2013; Nie et al., 2015). An excess of certain minerals can interfere with the absorption of other minerals (Robbins, 1983) and even cause diseases (Calvo & Tucker, 2013; Khan et al., 2016). Thus, the mineral balance strategy influences primates’ food choice, as revealed by study on proboscis monkeys (*Nasalis larvatus*) who select foods containing high P and low Ca (Yeager et al., 1997). Therefore, Assamese macaques choose young leaves of *B. saxatilis* that are rich in P to help them achieve a mineral balance in Ca‐rich limestone forests. Similarly, sympatric primates in limestone forests are known to lick the bare rock surface, which may also be a strategy to achieve mineral balance (Li, Wei, & Rogers, 2003; Zhou, Wei, Li, Huang, & Luo, 2006), as carbonate rocks contain many different trace minerals (Yuan, 1994).

The young leaves of *B. saxatilis* are very rich in protein, even higher than the average content of all the staple foods, which could be the key determinant for the Assamese macaques to consume throughout a whole year. Specifically, the water content in young bamboo leaves is higher than that of other nonfood items, which may effectively meet their water requirements in limestone forests where water is in shortage, suggesting that high water content may be another driving factor for their heavy dependence on these young bamboo leaves. Furthermore, the abundance of P in *B. saxatilis* could contribute to maintain the mineral balance for Assamese macaques in the high‐Ca limestone forests. It is worth also noting that the tannin content in young leaves and mature leaves of *B. saxatilis* is extremely low, ranging from 0.004% to 0.03% of dry matter, respectively. This content level is far below other nonbamboo foods, such as *T. brevistylum* and *C. ochlandra* contain 7.4% tannin and 3.4% tannin, respectively. In fact, many bamboos have extremely low or undetectable levels of tannins in their young parts (Wang et al., 2017). Meanwhile, tannin was not included into the highly supported model when examining the effects of nutrient contents on macaques’ food choice, suggesting that tannin had no significant effect on the food choice of Assamese macaques. Therefore, high protein, high water content, high minerals, and low tannin content meet the nutritional needs of Assamese macaques in the limestone forest, enabling them to maintain a bamboo‐leaf‐based diet.

In summary, we conclude that Assamese macaques living in limestone forests prioritize protein‐rich food items, consistently with the protein maximization model. Additionally, water and mineral balance was also considered in their food choices in the unusual limestone habitat. Moreover, young leaves of *B. saxatilis* contained more water, crude protein, P, and less tannin than nonfood items. The nutrient content of young leaves of *B. saxatilis* could meet the primates’ need in protein and water, while enabling them to maintain their mineral balance. This dietary preference is an adaptation to the unique karst habitat, consequently facilitating to maintain the primates’ bamboo‐leaf‐diet in the limestone forest. Our findings confirm the effects of nutrient contents in food choice of Assamese macaques, highlighting the importance of the nutrient contents in maintaining their bamboo‐based diet and the need to increase the knowledge on their nutrition strategy adapted to the bamboo‐dominated diet inhabiting the unique limestone habitat.

## CONFLICT OF INTERESTS

None declared.

## AUTHOR CONTRIBUTION

Yuhui Li: Formal analysis (equal); Writing‐original draft (equal). Guangzhi Ma: Conceptualization (equal); Methodology (equal). Qihai Zhou: Funding acquisition (equal); Investigation (equal); Methodology (equal). Youbang Li: Formal analysis (equal); Funding acquisition (equal); Methodology (equal); Writing‐review & editing (equal). Zhonghao Huang: Conceptualization (equal); Funding acquisition (equal); Investigation (equal); Methodology (equal); Writing‐review & editing (equal). 

## Data Availability

All data are available in the open figshare repository, and the link to the data is https://doi.org/10.6084/m9.figshare.12016815.v1.
